# Integrin αvβ6 Expression in Parathyroid Adenomas and Adjacent Non-Neoplastic Parathyroid Tissue: Immunohistochemical Rationale for Integrin αvβ6-Targeted PET Imaging

**DOI:** 10.3390/cancers18183026

**Published:** 2026-09-17

**Authors:** Amro Tuffaha, Wael Hananeh, Mario Liese, Dai Que Vu, Muin Tuffaha, Michael Starke

**Affiliations:** 1 Medical Clinic, Medical University Lausitz-Carl-Thiem, 03048 Cottbus, Germany; 2 Department of Veterinary Pathology and Public Health, Faculty of Veterinary Medicine, University of Science and Technology, Irbid 22110, Jordan; 3 Surgical Clinic, Oder Spree Krankenhaus, 15848 Beeskow, Germany; 4 Surgical Clinic, Medical University Lausitz-Carl-Thiem, 03048 Cottbus, Germany; 5 Institute of Pathology, Medical University Lausitz-Carl-Thiem, 03048 Cottbus, Germany; 6 Department of Nuclear Medicine, Medical University Lausitz-Carl-Thiem, 03048 Cottbus, Germany; m.starke@mul-ct.de

**Keywords:** primary hyperparathyroidism, parathyroid adenoma, integrin αvβ6, immunohistochemistry, ^68^Ga-Trivehexin, PET/CT, molecular imaging

## Abstract

Background: Accurate preoperative localization of hyperfunctioning parathyroid tissue is essential for focused parathyroid surgery; however, localization remains challenging in a subset of patients with small, ectopic, multiglandular, or conventionally occult lesions. ^68^Ga-Trivehexin targets integrin αvβ6 and has shown promising clinical performance in primary hyperparathyroidism; however, the immunohistochemical distribution of the integrin αvβ6 in parathyroid adenomas and non-neoplastic parathyroid tissue remains insufficiently characterized. Methods: Integrin αvβ6 expression was assessed immunohistochemically in 56 surgically resected parathyroid adenomas. Adjacent morphologically non-neoplastic parathyroid tissue was available in 51 cases and served as a paired comparator. Only membranous staining was scored. Staining intensity was categorized as 0, 1+ (weak), 2+ (moderate), or 3+ (strong), and the percentage of cells at each intensity was recorded. A weighted integrin αvβ6 expression score was calculated as (1 ×% weak) + (2 ×% moderate) + (3 ×% strong), yielding a range of 0–300. Paired comparisons were performed, and correlations with adenoma dimensions were assessed. Results: Forty-five of 56 adenomas (80.4%) and 48 of 51 adjacent non-neoplastic samples (94.1%) met the predefined positivity criterion. The median integrin αvβ6-positive cell fraction and weighted score were 42.5% (interquartile range (IQR) 9.5–90.0%) and 60 (IQR, 10–140), respectively. The corresponding values in adjacent tissue were 50% (IQR, 20–80%) and 60 (IQR, 30–115), respectively. Neither the weighted score nor the total positive-cell fraction differed significantly in paired analyses (Wilcoxon *p* = 0.902 and *p* = 0.442, respectively). Categorical positivity also did not differ significantly (exact McNemar *p* = 0.065). Crucially, the distribution of staining intensity differed between the compartments: a strong (3+) component was present in 19/56 adenomas (33.9%), but in only 3/51 adjacent samples (5.9%). In exploratory paired analysis, the proportion of strongly stained cells was higher in adenomas (Wilcoxon *p* = 0.0015). Integrin αvβ6 expression was not significantly associated with adenoma dimension. Conclusions: Integrin αvβ6 is a common membranous phenotype in both parathyroid adenomas and adjacent non-neoplastic parathyroid tissue and therefore should not be regarded as adenoma-specific. Nevertheless, adenomas more frequently contain strongly integrin αvβ6-positive cell populations, indicating a redistribution of expression intensity rather than a simple increase in overall positivity or weighted expression. These findings provide tissue-level support for integrin αvβ6-targeted parathyroid imaging and offer a plausible histopathological framework for the encouraging clinical performance of ^68^Ga-Trivehexin PET/CT. Prospective, lesion-matched studies integrating immunohistochemistry, PET metrics, lesion volume, operative findings, and histological confirmation are now required to validate these observations.

## Simple Summary

Preoperative localization of hyperfunctioning parathyroid glands can be challenging, particularly when lesions are small, ectopic, multiglandular, or occult on conventional imaging. The novel PET tracer ^68^Ga-Trivehexin targets integrin αvβ6 and has shown promising results for detecting hyperfunctioning parathyroid tissue; however, the prevalence of its molecular target in parathyroid lesions has remained unclear. We evaluated integrin αvβ6 immunohistochemically in 56 surgically resected parathyroid adenomas and, where available, adjacent non-neoplastic parathyroid tissue. Integrin αvβ6 was frequently expressed in both adenomatous and non-neoplastic tissue; therefore, it is not tumor-specific; however, strong (3+) membranous staining was markedly more frequent in adenomas. These findings provide a tissue-level mechanistic basis for αvβ6-targeted parathyroid imaging and support further imaging–pathology correlation studies aimed at refining preoperative localization strategies.

## 1. Introduction

Primary hyperparathyroidism (PHPT) is most commonly caused by a solitary parathyroid adenoma. Parathyroidectomy is the definitive treatment for appropriate surgical candidates, and accurate preoperative localization is particularly important when a focused or minimally invasive approach is planned [1,2]. Although ultrasonography, ^99m^Tc-sestamibi (methoxyisobutylisonitrile, MIBI) scintigraphy/single photon emission computed tomography/computed tomography (SPECT/CT), four-dimensional CT, and increasingly fluorocholine (^18^F-fluorocholine) positron emission tomography/computed tomography (PET/CT) can localize many hyperfunctioning glands, clinically relevant localization failures remain, particularly in patients with small lesions, ectopic glands, multiglandular disease, or discordant imaging findings [3,4,5,6,7,8].

Integrin αvβ6 is a cell-surface heterodimer composed of the αv and β6 subunits. Because the β6 subunit is restricted to integrin αvβ6, it is commonly used as a tissue surrogate for the presence of the heterodimeric receptor [9]. Integrin αvβ6 has attracted interest as an imaging and theranostic target because it is accessible at the plasma membrane and can be upregulated during epithelial remodeling, fibrosis, and a range of neoplasms [10,11,12].

**Figure 1:**
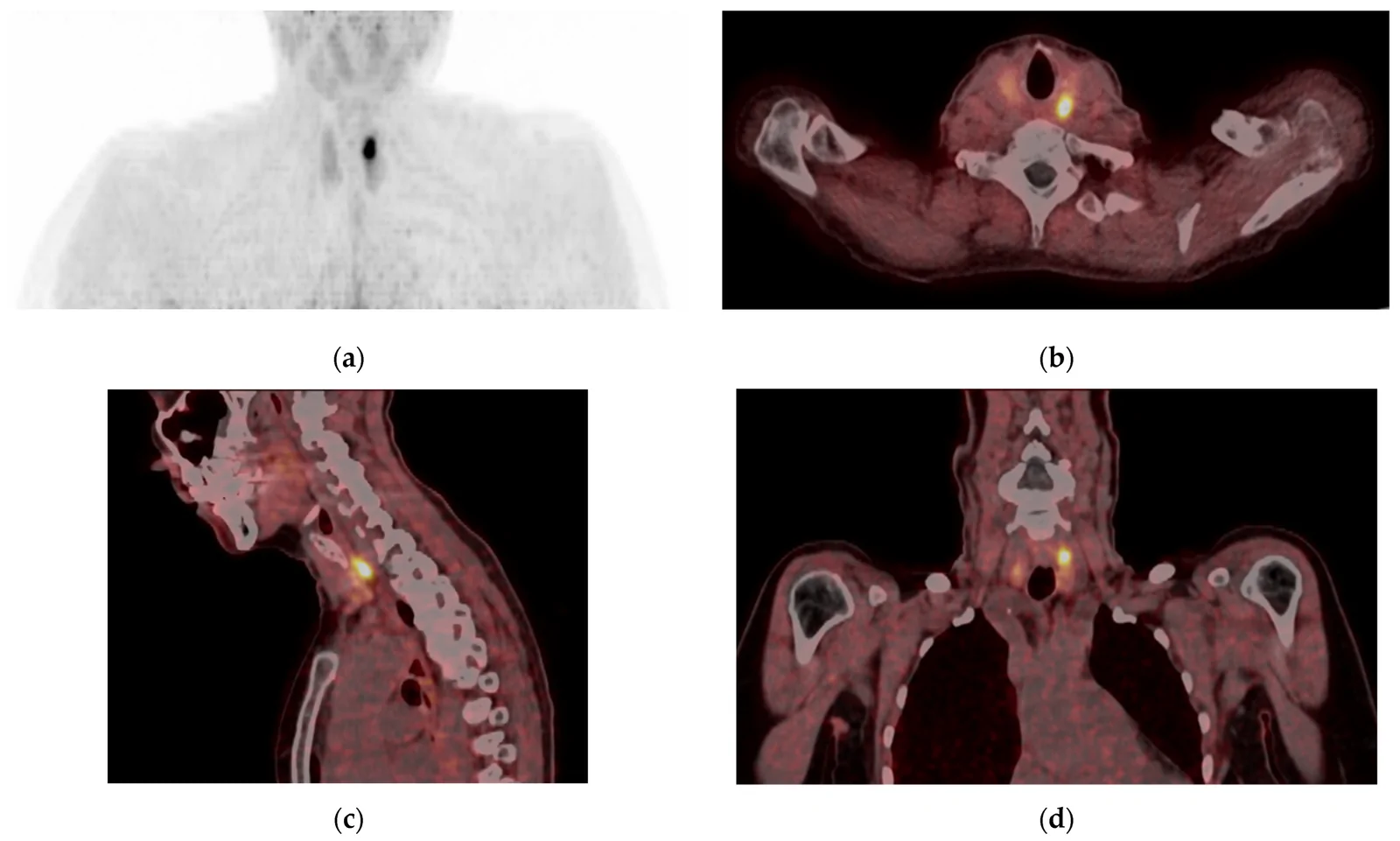
(**a**) Maximum-intensity-projection (MIP) image of ^68^Ga-Trivehexin PET acquired 30 min post-injection in a 65-year-old woman with hypercalcemia and hyperparathyroidism. Focal intense tracer uptake is seen in the upper-left parathyroid region, corresponding to the suspected parathyroid adenoma; (**b**) transverse fused ^68^Ga-Trivehexin PET/CT image acquired 30 min post-injection showing a focal area of intense tracer uptake is identified in the upper-left parathyroid region, corresponding to the suspected parathyroid adenoma; (**c**) sagittal fused ^68^Ga-Trivehexin PET/CT image acquired 60 min post-injection showing persistent focal intense tracer uptake is seen in the upper-left parathyroid region, corresponding to the suspected parathyroid adenoma; (**d**) coronal fused ^68^Ga-Trivehexin PET/CT image acquired 60 min post-injection showing persistent focal intense tracer uptake is demonstrated in the upper-left parathyroid region, corresponding to the suspected parathyroid adenoma.

^68^Ga-Trivehexin is an integrin αvβ6-selective positron emission tomography (PET). A 2024 clinical study of 13 patients with primary hyperparathyroidism (PHPT) reported a lesion-based detection rate of 94.1% for ^68^Ga-Trivehexin PET/CT compared with 58.8% for ^99m^Tc-MIBI imaging, and a larger study in 2026 reported a sensitivity of 97.3% against a composite reference standard, with a particular value in MIBI-negative patients [13,14,15]. Our imaging observations in 11 cases were consistent with those reported in these studies (Figure 1a–d). Although clinically provocative, these imaging findings do not establish the tissue basis of tracer uptake in isolation. Therefore, systematic characterization of integrin αvβ6 expression in parathyroid adenomas and paired non-neoplastic parathyroid tissue is important for two reasons. First, demonstration of membranous integrin αvβ6 expression in adenoma cells establishes the tissue-level molecular substrate required for integrin αvβ6-directed ligand binding. Second, comparison with non-neoplastic parathyroid tissue helps determine whether integrin αvβ6 expression is associated with adenomatous transformation or instead represents a broader parathyroid epithelial phenotype.

The primary aim of this pathology-led study was to characterize the extent and intensity of membranous integrin αvβ6 expression in parathyroid adenomas and adjacent morphologically non-neoplastic parathyroid tissue. Secondary aims were to compare paired weighted expression scores and positive-cell fractions, examine the distribution of staining intensities, explore patterns across histological subtype patterns, and assess whether integrin αvβ6 expression is associated with adenoma size. This study was not designed to evaluate the diagnostic accuracy of ^68^Ga-Trivehexin PET/CT.

## 2. Materials and Methods

### 2.1. Study Design, Case Selection and Tissue Samples

This retrospective observational study included archived formalin-fixed, paraffin-embedded (FFPE) tissue from 56 surgically resected, histologically confirmed parathyroid adenomas. Adjacent morphologically non-neoplastic parathyroid tissue was available in 51 cases and served as a paired comparator. It should be noted that tissues immediately adjacent to an adenoma may not be biologically identical to truly normal parathyroid gland tissue distant from the lesion; microenvironment influences related to the adenoma cannot be excluded. The cohort comprised 43 conventional adenomas, 9 oncocytic adenomas, 3 water-clear cell adenomas, and 1 lipoadenoma. The recorded adenoma dimensions ranged from 8 to 45 mm (median, 16 mm; mean, 18.6 ± 7.8 mm). All cases were retrieved from the archive of the Institute of Pathology at the Medical University Lausitz-Carl-Thiem, Germany, covering the period from January 2023 to June 2026. All tissue samples consisted of all available diagnostic specimens.

Hematoxylin and eosin-stained sections were reviewed by a pathologist to confirm the diagnosis, verify the presence of adjacent non-neoplastic parathyroid tissue where available, and assess histomorphology. Parathyroid origin was supported by the diagnostic immunophenotype, including the expression of parathyroid hormone and GATA-binding protein 3 (GATA3). Tumors were classified according to the 5th edition of the World Health Organization (WHO) Classification of Endocrine and Neuroendocrine Tumors [16,17].

### 2.2. Immunohistochemistry

Serial 1 µm thick sections were cut from FFPE tissue blocks and mounted on positively charged glass slides. After deparaffinization and rehydration, heat-induced antigen retrieval was performed using Tris-EDTA buffer, pH 9.0. Endogenous peroxidase activity was blocked before incubation with a rabbit monoclonal antibody against integrin αvβ6 (clone E4M9P; Cell Signaling Technology, Danvers, MA, USA) at a dilution of 1:200, anti-GATA3 mouse monoclonal antibody (clone L50-823, Cell Marque, Rocklin, CA, USA) at a dilution of 1:300, and anti-Parathyroid Hormone (PTH) mouse monoclonal antibody (clone MRQ-31, Cell Marque, Rocklin, CA, USA) at a dilution of 1:400. Immunoreactivity was visualized using a two-step horseradish peroxidase/diaminobenzidine detection system, followed by hematoxylin counterstaining. Staining was performed under standardized conditions using identical reagents and an automated staining platform within a single experimental run to reduce inter-assay variability. Photomicrographs were captured, and the total magnifications are stated in the corresponding figure legends.

Negative controls were included in each staining run, consisting of serial sections incubated with an isotype-matched, non-immune rabbit IgG in place of the primary anti-integrin αvβ6 antibody at an equivalent concentration; sections of adjacent thyroid tissue, when present, served as an additional internal negative control, consistent with the consistently negative integrin αvβ6 staining subsequently observed in this tissue. After IHC slides were prepared, a pathologist performed a single-blind evaluation of the slides and the scores were recorded. During slide evaluation, the scoring pathologist had no access to any clinicopathological data. All evaluations were performed by a single pathologist. We acknowledge that semiquantitative assessment of staining intensity and percentage is subject to observer variability; however, interobserver reproducibility was not formally assessed in this study. This should be considered when interpreting the quantitative findings, particularly the distinction between weak, moderate, and strong staining.

### 2.3. Evaluation of Immunohistochemical Staining

Integrin αvβ6 expression was evaluated semi-quantitatively by assessing membranous staining intensity and the estimated percentage of positive tumor cells. Staining intensity was scored as 0, negative; 1, weak; 2, moderate; and 3, strong. The percentage of positive tumor cells was recorded in the evaluated tumor compartment. An H-score was calculated by multiplying the intensity by the percentage of positive tumor cells, resulting in a final score from 0 to 300. Mixed intensities were scored using the mean intensity value; for example, weak-to-moderate staining was scored as 1.5 and moderate-to-strong staining as 2.5. This convention was utilized solely for tumors in which two neighboring intensity grades were intermixed within the same membranous staining pattern and could not be allocated to a single discrete category; consequently, the resulting non-integer H-scores were preserved without rounding to maintain scoring resolution.

Cases were categorized as negative (H-score 0), weak/low expression (H-score 1–100), moderate expression (H-score 101–200), or strong/high expression (H-score 201–300) as previously described [18]. For the primary categorical positivity analysis, a sample was classified as positive if membranous staining of any intensity (weak, moderate, or strong) was present in more than 5% of cells in the evaluated compartment. Cases with ≤5% weakly positive cells were classified as negative. This threshold was applied consistently to both adenomas and adjacent no-neoplastic tissue. These categories are employed descriptively as a practical four-tier subdivision of the 0–300 H-score range; they do not denote a validated clinical threshold or therapeutic-relevance benchmark. Furthermore, the H-score corresponding to a tumor that amenable to integrin αvβ6-directed therapy remains to be determined. The intensity symbols (+/++/+++) and H-score categories are different scoring systems and should not be read as equivalent. The symbols record the raw staining intensity, whereas the H-score category is derived from the calculated intensity × percentage product.

### 2.4. Statistical Analysis

Continuous variables were summarized using the median and interquartile range (IQR) and, where informative, the mean ± standard deviation (SD). Paired differences between adenoma and adjacent non-neoplastic tissue in the weighted integrin αvβ6 expression score and total positive cell fraction were assessed using the Wilcoxon signed-rank test. Paired categorical positivity was compared using the exact McNemar’s test. Because the revised staining data indicated potentially important differences in intensity composition, exploratory paired Wilcoxon analyses were also performed for the percentages of weakly, moderately, and strongly stained cells; these analyses were considered secondary and hypothesis-generating. Associations between adenoma dimensions and integrin αvβ6 expression were assessed using Spearman’s rank correlation, with Pearson’s correlation reported as a complementary analysis. Two-sided *p*-values were reported, and *p* < 0.05 was considered statistically significant for the primary analyses.

## 3. Results

A total of fifty-six parathyroid adenomas and fifty-one adjacent non-neoplastic parathyroid tissues were evaluated for integrin αvβ6 expression by immunohistochemistry. Adequate tumor tissue was available for all cases. The cohort comprised 43 conventional adenomas, 9 oncocytic adenomas, 3 water-clear cell adenomas, and 1 lipoadenoma. The recorded adenoma dimensions ranged from 8 to 45 mm (median, 16 mm; mean, 18.6 ± 7.8 mm). Five cases lacked an appropriate adjacent tissue comparator on the immunostained sections.

Integrin αvβ6 was predominantly expressed on both parathyroid adenomas and adjacent non-neoplastic parathyroid tissues. Table 1 shows detailed case-level data on adenoma subtype, lesion dimension, and integrin αvβ6 staining intensity and distribution in adenomatous and adjacent non-neoplastic parathyroid tissue.

### 3.1. Integrin αvβ6 Expression in Parathyroid Adenomas

Using the predefined categorical criterion, 45 of 56 adenomas (80.4%) were integrin αvβ6-positive. The median proportion of integrin αvβ6-immunoreactive adenoma cells was 42.5% (IQR, 9.5–90.0%), with a mean of 47.4 ± 38.6%. The median weighted integrin αvβ6 expression score was 60 (IQR, 10–140), with a mean of 78.9 ± 72.5.

**Figure 2:**
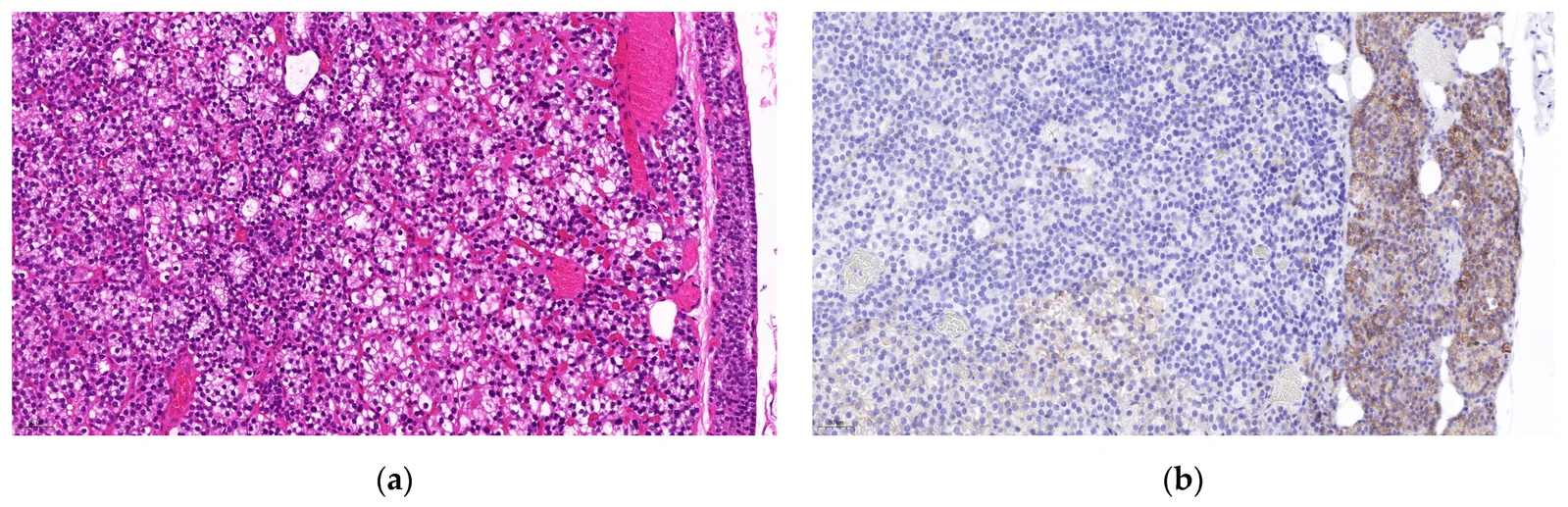
(**a**) A parathyroid adenoma with an adjacent rim of atrophic normal parathyroid tissue; H&E. (**b**) Integrin αvβ6 immunohistochemical staining of the same section shows very weak membranous integrin αvβ6 expression in approximately 20% of the adenoma cells, whereas the remaining adenoma cells lack integrin αvβ6 expression. The adjacent normal parathyroid tissue shows diffuse, moderate membranous integrin αvβ6 expression, (scale 50 µm); IHC.

**Figure 3:**
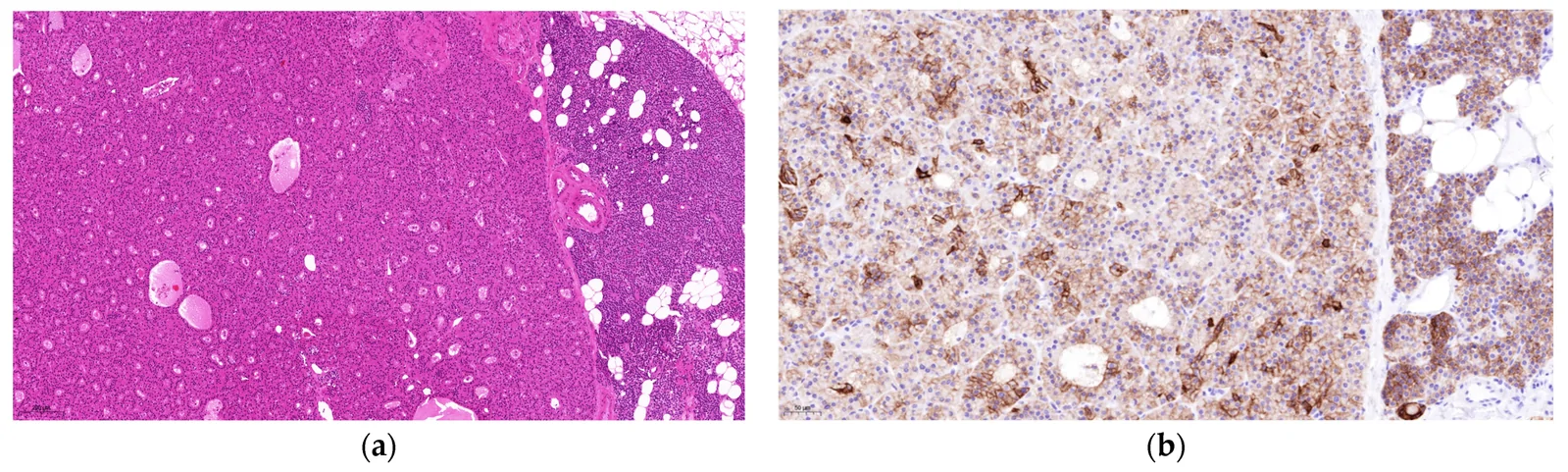
(**a**) A parathyroid adenoma with adjacent normal parathyroid tissue (scale 50 µm); H&E. (**b**) Integrin αvβ6 immunohistochemical staining of the same section shows moderate membranous integrin αvβ6 expression in approximately 90% of the adenoma cells, with strong expression observed in approximately 10% of the adenoma cells. The adjacent normal parathyroid tissue shows diffuse, moderate to strong membranous integrin αvβ6 expression, (scale 50 µm); IHC.

**Figure 4:**
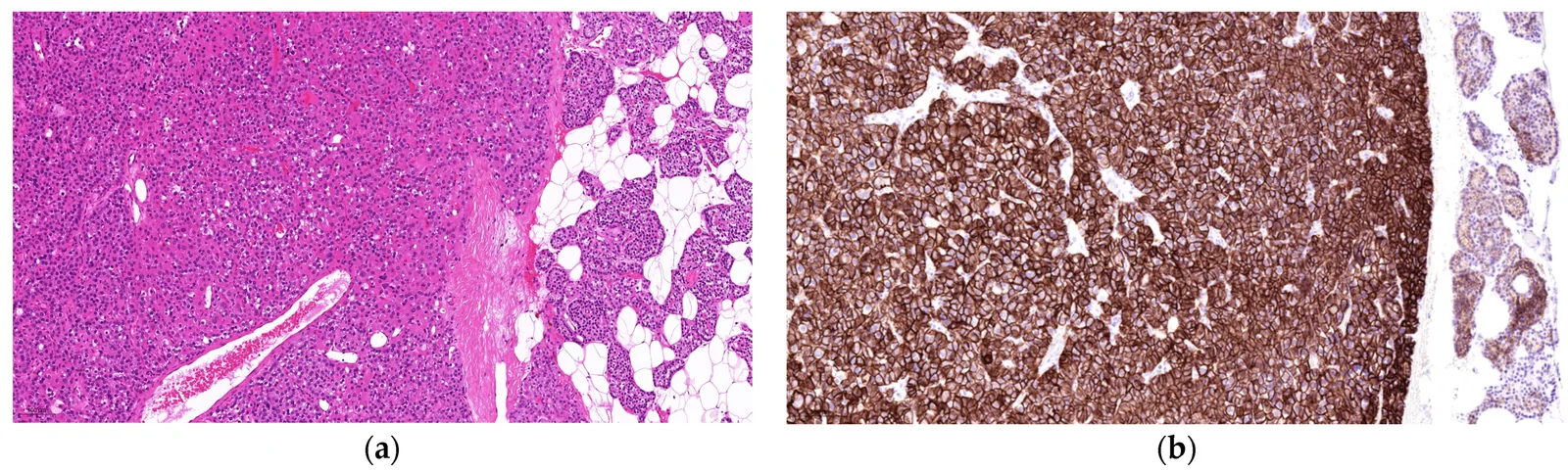
(**a**) A parathyroid adenoma with adjacent atrophic, normal parathyroid tissue (left) (scale 100 µm); H&E. (**b**) Integrin αvβ6 immunohistochemical staining of the same section shows very strong membranous integrin αvβ6 expression in 100% of the adenoma cells. The adjacent normal parathyroid tissue shows weak membranous integrin αvβ6 expression (scale 100 µm); IHC.

Expression was heterogeneous both between and within adenomas. A weak (1+) component was present in 43/56 cases (76.8%) (Figure 2a,b), a moderate (2+) component in 41/56 (73.2%) (Figure 3a,b), and a strong (3+) component in 19/56 (33.9%) (Figure 4a,b). The mean proportions of weakly, moderately, and strongly stained adenoma cells were 21.9%, 19.7%, and 5.9%, respectively. When cases were classified according to the highest staining intensity present, 7 adenomas were completely negative, 8 had a maximum intensity of 1+, 22 of 2+, and 19 of 3+. Thus, the adenoma cohort included a substantial subgroup with focal-to-extensive strong membranous integrin αvβ6 expression.

### 3.2. Integrin αvβ6 Expression in Adjacent Non-Neoplastic Parathyroid Tissues

Among the 51 evaluable adjacent samples, 48 (94.1%) met the positivity criterion. The median positive-cell fraction was 50% (20–80%), with a mean of 52.1 ± 30.9%. The median weighted expression score was 60 (30–115), with a mean of 78.5 ± 57.3.

A weak component was present in 36/51 samples (70.6%) (Figure 4a,b), a moderate component in 31/51 (60.8%) (Figure 2a,b), and a strong component in only 3/51 (5.9%) (Figure 3a,b). The mean proportions of weakly, moderately, and strongly stained cells were 26.6%, 24.5%, and 1.0%, respectively. By maximum intensity, 20 samples showed only 1+ staining, 28 reached 2+, and 3 reached 3+; none of the evaluable adjacent samples was completely unstained, although three were categorized as negative because staining was restricted to ≤5% weakly positive cells.

### 3.3. Paired Comparison of Adenoma and Adjacent Non-Neoplastic Parathyroid Tissue

Fifty-one cases were available for direct paired analysis. The median weighted integrin αvβ6 expression score was 60 in both compartments. There was no evidence of a systematic paired difference in weighted score (Wilcoxon *p* = 0.902): the score was higher in adenoma in 24 cases, higher in adjacent tissue in 24 cases, and identical in 3 cases. The total fraction of integrin αvβ6-immunoreactive cells also did not differ significantly (Wilcoxon *p* = 0.442).

Categorical positivity was numerically lower in adenomas than in adjacent tissue (80.4% overall in adenomas; 94.1% among evaluable adjacent samples). In the 51 paired cases, there were two adenoma-positive/adjacent-negative pairs and nine adenoma-negative/adjacent-positive pairs; the exact McNemar’s test did not reach conventional statistical significance (*p* = 0.065).

The most notable difference was the distribution of the staining intensity rather than the total expression. A strong (3+) component was observed in 33.9% of adenomas but only in 5.9% of adjacent samples. In exploratory paired analysis, the percentage of strongly stained cells was higher in adenomas (Wilcoxon *p* = 0.0015), whereas paired differences in weak and moderate staining were not significant (*p* = 0.361 and *p* = 0.163, respectively). Accordingly, the revised data support a shift toward stronger membranous staining in a subset of adenomas without a corresponding increase in the global weighted score or the total positive cell fraction. The comparison of weak, moderate and strong staining fractions, including the strong-staining analysis above, constitutes a post hoc exploratory analysis; no correction for multiple comparisons was applied. These results should be used only to generate hypotheses for future studies and must not be treated as confirmatory findings.

### 3.4. Histological Subtype Patterns

Exploratory, descriptive analysis suggested heterogeneity among histological variants. Conventional adenomas (*n* = 43) had a median weighted score of 60 and median positive-cell fraction of 40%. Oncocytic adenomas (*n* = 9) had a median score of 60 and median positive-cell fraction of 50%. The three water-clear cell adenomas showed lower values (median score 20; median positive-cell fraction 15%), whereas the single lipoadenoma had a score of 110 and a positive cell fraction of 80%. Because of the very small numbers of non-conventional variants (water-clear cell, *n* = 3; lipoadenoma, *n* = 1), these observations are purely descriptive and do not support statistical interference or subtype-specific conclusions. Detailed case-level data by subtype are provided in Supplementary Table S1.

### 3.5. Relationship Between Adenoma Size and Integrin αvβ6 Expression

Adenoma dimension was not significantly associated with the weighted integrin αvβ6 expression score or the fraction of integrin αvβ6-positive cells. The Spearman coefficients were ρ = 0.154 (*p* = 0.256) for adenoma dimension versus weighted score and ρ = 0.158 (*p* = 0.244) for dimension versus positive cell fraction. Pearson analyses were similarly non-significant (r = 0.180, *p* = 0.185 and r = 0.196, *p* = 0.147, respectively). These findings indicate that larger adenomas did not systematically show greater cellular integrin αvβ6 expression.

## 4. Discussion

This study provides an immunohistochemical characterization of integrin αvβ6 expression in a relatively large series of parathyroid adenomas with paired adjacent non-neoplastic tissue and has direct implications for the interpretation of integrin αvβ6-targeted PET. The case-level data support four principal observations. First, membranous integrin β6 expression is common in parathyroid adenomas and is present, by the study definition, in approximately four-fifths of lesions. Second, integrin αvβ6 is not adenoma-specific, because adjacent parathyroid tissue is also frequently positive. Third, while exploratory analysis suggests a redistribution of staining intensity, with strong membranous staining substantially more frequent in adenomas. Fourth, cellular integrin αvβ6 expression is not significantly associated with adenoma size.

The β6 subunit partners with αv to form integrin αvβ6, and membranous integrin αvβ6 immunoreactivity provides tissue-level evidence of the molecular component required for αvβ6-directed ligand binding. This is directly relevant to ^68^Ga-Trivehexin, an integrin αvβ6-selective PET tracer. The published clinical literature has evolved rapidly: an initial 2024 PHPT series reported the detection of 16 of 17 hyperfunctioning lesions with ^68^Ga-Trivehexin, including additional lesions not visualized by MIBI, and a 2026 two-center analysis reported 97.3% sensitivity against a composite reference standard, with frequent positivity in MIBI-negative patients [13,14]. The present study provides a plausible pathological substrate for these imaging observations by demonstrating membrane-associated integrin αvβ6 expression in most adenomas.

However, immunohistochemical target expression and imaging performance should remain conceptually distinct from each other. The present cohort was not analyzed using matched lesion-level Trivehexin PET parameters; therefore, this study could not establish the sensitivity, specificity and superiority over other modalities, or the quantitative relationship between integrin αvβ6 staining and tracer uptake. Rather, its contribution is mechanistic and translational, as it establishes that the target is commonly present and characterizes its histological distribution.

The high frequency of integrin αvβ6 expression in adjacent morphologically non-neoplastic tissue is central to the biological interpretation. Indeed, categorical positivity was numerically more frequent in adjacent tissue than in adenomas, and paired weighted scores were essentially identical. Thus, terms such as “adenoma-specific marker” or “overexpression in adenoma” are not supported by these data. A more accurate interpretation is that integrin αvβ6 represents a broader parathyroid epithelial phenotype in which the distribution of staining intensity changes in at least a subset of adenomas. This distinction is important for both pathology and nuclear medicine. At the microscopic level, non-neoplastic parathyroid cells express the receptor; however, at the whole-organ level, a normal gland contains far less receptor-bearing tissue than an adenoma and often contains substantial adipose tissue. The PET signal depends not only on receptor expression per cell but also on the absolute number and density of target-positive cells, lesion volume, perfusion, ligand delivery and accessibility, internalization, scanner resolution, background activity, and partial-volume effects. Consequently, frequent integrin αvβ6 expression in normal-sized parathyroid tissue is not incompatible with the focal visualization of an adenoma.

This physiological background expression carries direct clinical implications that warrant explicit consideration. A hyperplastic or adenomatous second gland in genuine multiglandular disease would be expected to combine a larger tissue volume with comparable or higher receptor density, so it should, in principle, still generate a detectable focal signal rather than being obscured by background; the biophysical explanation above (limited tissue mass and substantial adipose content in small, physiologically normal glands) offer a plausible mechanism for why single, focal uptake is nonetheless the dominant pattern reported clinically. Nonetheless, on the basis of immunohistochemistry alone, we cannot exclude a theoretical risk that mildly enlarged or multifocally active, non-adenomatous parathyroid tissue could generate a false-positive or equivocal PET signal, potentially contributing to overdiagnosis or multiglandular hyperplasia or unnecessary escalation from focused, minimally invasive adenectomy to bilateral neck exploration. Mitigating this risk in practice will likely require semiquantitative interpretation criteria (e.g., relative uptake or SUV rations between candidate glands rather than binary positive/negative reads), integration with biochemical and other localizing studies, and prospective, lesion-matched imaging–pathology correlation to define clinically validated uptake thresholds. This histological findings of high background positivity therefore motivates the development of such quantitative interpretation criteria; it does not, by itself, contradict the high clinical sensitivity already reported for ^68^Ga-Trivehexin PET/CT.

The most informative revised finding is the disparity in strong staining. Approximately one-third of adenomas contained a 3+ component, compared to only a small minority of adjacent samples. The proportion of strongly stained cells was significantly higher in the exploratory paired analysis despite similar total positive-cell fractions and weighted scores. This finding illustrates why a simple positive/negative classification may obscure biologically relevant differences in receptor-expression architecture.

For receptor-targeted imaging, a strongly positive tumor cell population can increase local ligand binding and lesion-to-background contrast even when the global H-score-like metric is not increased. Conversely, widespread weak or moderate expression in a very small normal gland may result in limited total tracer retention. Future imaging–pathology studies should therefore correlate PET uptake not only with a single weighted score but also with the fraction of 2+/3+ cells, the strong-staining fraction, lesion cellularity, and lesion volume. A composite measure of “receptor-positive tissue burden” may prove more informative than staining intensity alone. Receptor expression at histology provides only one compartment of the determinants of PET contrast. Tracer delivery, plasma protein bindings, internalization, washing out kinetics, and background activity may vary substantially over time, and optimal timing of PET acquisition is therefore an important transitional consideration that cannot be inferred for immunohistochemistry alone. Emerging technologies such as long-axial field-of-view (LAFOV) PET systems may facilitate dynamic studies with improved signal-to-noise rations and temporal sampling, potentially refining the characterization of Trivehexin pharmacokinetics and improving lesion-to-background quantification [19,20,21].

The absence of a significant correlation between adenoma size and cellular integrin αvβ6 expression suggested that target expression is not simply a feature of larger lesions. This finding supports the biological feasibility of imaging small adenomas, including ectopic lesions, which remain clinically challenging to localize. Nevertheless, preserved receptor expression in a small lesion does not guarantee PET visibility, because spatial resolution and partial-volume effects can reduce apparent uptake. Future studies should therefore distinguish between two separate questions: whether integrin αvβ6 expression is maintained in small lesions and whether lesion size influences PET detectability or quantitative SUV.

In contrast to receptor-targeted tracers, ^99m^Tc-sestamibi localization depends on biological characteristics of the parathyroid lesion, particularly mitochondrial abundance and cellular composition, including the proportion of mitochondria-rich oxyphil cells [22,23,24]. ^18^F-fluorocholine PET/CT has also emerged as a high-performance modality with substantial clinical availability and serves as an important contemporary comparator when evaluating novel molecular tracers such as ^68^Ga-Trivehexin [25,26]. The two strategies mitochondrial/fluorocholine-based and integrin-targeted-are therefore biologically distinct rather than redundant. This mechanistic difference may help explain why Trivehexin-positive lesions can occur in patients with negative or discordant MIBI studies, as reported clinically [13,14]. A lesion-matched study integrating histomorphology, integrin αvβ6 expression, conventional imaging, and Trivehexin PET would therefore be valuable and represents the focus of our next project.

The descriptive subtype analysis suggests that β6 expression may vary among parathyroid adenoma morphologies, with relatively low scores in the three water-clear cell adenomas and substantial expression in most oncocytic adenomas. The subgroup sizes were too small for inferential testing; therefore, these observations should be considered hypothesis-generating. In particular, the water-clear cell (*n* = 3) and lipoadenoma (*n* = 1) subgroups are far too small to support any conclusion that expression genuinely differs by subtype, and these figures should not read as a validated subtype comparison. Nevertheless, they support the broader concept that cellular phenotypes may influence receptor-targeted imaging and should be recorded prospectively in future imaging–pathology cohorts.

The demonstration of integrin αvβ6 expression in non-neoplastic parathyroid tissue is also relevant when considering integrin αvβ6-directed radioligand therapy for malignancies that express this target protein. Similar to normal pituitary endocrine cells, normal parathyroid cells may represent a potential on-target site for tracer accumulation and, consequently, radiation exposure. Therefore, therapeutic approaches using β-emitting, α-emitting, or other high-linear energy-transfer radionuclides should consider the possibility of parathyroid irradiation and its potential effects on parathyroid function and calcium-parathyroid hormone homeostasis [27,28]. This concern remains theoretical in the present study, particularly because comparable integrin αvβ6 expression was also observed in histologically normal parathyroid glands incidentally identified in five thymectomy specimens, whereas adjacent normal thyroid tissues were consistently negative for integrin αvβ6 expression. It should be emphasized that the present study did not include radiation dosimetry, in vivo animal biodistribution data, or clinical radioligand therapy outcome data; the discussion above is therefore an extrapolation from immunohistochemical findings alone and should be read as a theoretical, hypothesis-generating consideration rather than a dosimetric or clinical claim. These findings should not be interpreted as evidence of clinical toxicity. Rather, they identified the parathyroid glands as a potential organ at risk that should be considered in dosimetric assessments and be prospectively monitored for possible functional effects during integrin αvβ6-targeted radioligand therapy [29,30]. A similar precautionary approach has been proposed for the pituitary gland in the context of integrin αvβ6-targeted radioligand therapy, given its physiological uptake of αvβ6-targeted tracers [31].

The major strengths of this study include the use of case-level semiquantitative immunohistochemistry, systematic recording of both staining extent and intensity, and a paired design that directly assesses whether integrin αvβ6 expression is specific to parathyroid adenomatous tissue or is also characteristic of non-neoplastic parathyroid tissue. Recalculation of the results table further indicates that the principal difference between adenomatous and non-neoplastic parathyroid tissues lies in the distribution of staining intensity rather than a global increase in integrin αvβ6 expression.

This study has several limitations should be acknowledged. First, immunohistochemistry provides a semiquantitative assessment of integrin αvβ6 expression and does not directly measure absolute receptor density, ligand-binding affinity, or functional receptor occupancy. Second, non-conventional parathyroid adenoma subtypes (water-clear cell adenoma, *n* = 3; lipoadenoma, *n* = 1) were infrequently represented in the cohort, precluding meaningful and statistically robust comparisons between histological subtypes; findings for these subgroups are descriptive only and are reported in Supplementary Table S1. Third, the staining intensity analyses were exploratory and should therefore be interpreted cautiously and validated in larger independent cohorts. Fourth, the paired comparisons of weak, moderate, and strong staining fractions, including the exploratory strong-staining results, were post hoc and were not corrected for multiple testing; these results are hypothesis-generating and should not be treated as a confirmatory. Fifth, this study did not include radiation dosimetry, in vivo animal biodistribution data, or clinical radioligand therapy outcome data, so the discussion of potential parathyroid irradiation during integrin αvβ6-targeted therapy is theoretical rather than a dosimetric or clinical finding. Finally, immunohistochemical expression alone does not establish whether the detected receptor is functionally accessible for tracer binding or therapeutic targeting.

## 5. Conclusions

Integrin αvβ6 is frequently expressed on the membranes of parathyroid adenoma cells, but it is also commonly present in adjacent morphologically non-neoplastic parathyroid tissue and therefore is not an adenoma-specific marker. The case-level data do not demonstrate higher overall integrin αvβ6 positivity, a higher positive-cell fraction, or a higher weighted expression score in adenomas. Instead, they reveal a distinct intensity architecture, with strong (3+) membranous staining occurring substantially more often in adenomas than in adjacent tissue.

This expression pattern provides a plausible immunohistopathological basis for integrin αvβ6-targeted imaging and offers a mechanistic explanation for focal ^68^Ga-Trivehexin uptake in parathyroid adenomas despite integrin αvβ6 expression in non-neoplastic parathyroid cells. Emerging imaging studies further support the potential clinical value of ^68^Ga-Trivehexin, while the present immunohistopathological findings provide a rationale for investigating whether integrin αvβ6-targeted PET may offer complementary or improved localization compared with conventional imaging approaches. Prospective, lesion-matched studies integrating integrin αvβ6 immunohistochemistry, the fraction of strongly positive cells, lesion cellularity and volume, quantitative PET parameters, operative localization, and histological confirmation are warranted to determine whether integrin αvβ6-targeted PET can improve preoperative localization, particularly in patients with small, ectopic, multiglandular, or conventionally occult parathyroid lesions.

## Figures and Tables

**Table 1:** Immunohistochemical expression of integrin αvβ6 in parathyroid adenoma subtypes, and adjacent non-neoplastic parathyroid tissue.

No.	Parathyroid Adenoma/Subtype	Dimension (mm)	Integrin αvβ6 Expression in Parathyroid Adenoma	Integrin αvβ6 Expression in Adjacent Parathyroid Tissue
1.	Conventional adenoma	15	+10% (H score = 10)	+5% (H score = 5)
2.	Conventional adenoma	28	+10% (H score = 10)	+20%; ++20% (H score = 60)
3.	Oncocytic adenoma	21	+20%; ++10% (H score = 40)	++70% (H score = 140)
4.	Conventional adenoma	12	+20%; ++5% (H score = 30)	+20%; ++20%; +++10 (H score = 90)
5.	Conventional adenoma	29	+10%; ++5% (H score = 20)	+10% (H score = 10)
6.	Conventional adenoma	25	+10%; ++20%; +++30% (H score = 140)	+50%; ++20% (H score = 90)
7.	Conventional adenoma	10	+20%; ++40%; +++20% (H score = 160)	++90% (H score = 180)
8.	Conventional adenoma	18	++20% (H score = 40)	NA
9.	Conventional adenoma	14	+80% (H score = 80)	+30% (H score = 30)
10.	Water-clear cell adenoma	18	+10%; ++5% (H score = 20)	++10% (H score = 20)
11.	Conventional adenoma	12	++1% (H score = 2)	++30% (H score = 60)
12.	Conventional adenoma	11	+50%; ++20%; +++20% (H score = 150)	+20% (H score = 20)
13.	Conventional adenoma	25	+40%; ++50% (H score = 140)	++20% (H score = 40)
14.	Oncocytic adenoma	23	+30%; ++40% (H score = 110)	+50%; ++50% (H score = 150)
15	Conventional adenoma	16	No expression in adenoma cells (H score = 0)	++30% (H score = 60)
16	Conventional adenoma	11	No expression in adenoma cells (H score = 0)	+20%; ++70% (H score = 160)
17	Conventional adenoma	15	+10%; ++10% (H score = 30)	+60%; ++30% (H score = 120)
18	Conventional adenoma	13	+3% (H score = 3)	+20% (H score = 20)
19	Conventional adenoma	33	+40%; ++20%; +++20% (H score = 140)	++70%; +++30% (H score = 230)
20	Conventional adenoma	45	+50%; ++40%; +++10% (H score = 160)	+20% (H score = 20)
21	Water-clear cell adenoma	15	No expression in adenoma cells (H score = 0)	+30% (H score = 30)
22	Conventional adenoma	17	+20%; ++20%; +++5% (H score = 75)	+30%; ++70% (H score = 170)
23	Oncocytic adenoma	24	+30%; ++30%; +++30% (H score = 180)	+50%; ++10% (H score = 70)
24	Conventional adenoma	15	No expression in adenoma cells (H score = 0)	+20%; ++20% (H score = 60)
25	Conventional adenoma	28	+30% (H score = 30)	++30% (H score = 60)
26	Conventional adenoma	11	+40%; ++20%; +++20% (H score = 140)	+50%; ++30% (H score = 110)
27	Conventional adenoma	18	+30%; ++20%; +++10% (H score = 100)	+60%; ++20% (H score = 100)
28	Conventional adenoma	14	+10%; ++10% (H score = 30)	+70%; ++20% (H score = 110)
29	Conventional adenoma	13	+30%; ++10% (H score = 50)	+20% (H score = 20)
30	Conventional adenoma	15	+5% (H score = 5)	+70%; ++10% (H score = 90)
31	Conventional adenoma	30	+40%; ++10% (H score = 60)	+40% (H score = 40)
32	Conventional adenoma	18	No expression in adenoma cells (H score = 0)	NA
33	Oncocytic adenoma	15	+10%; ++10%; +++5% (H score = 45)	+20% (H score = 20)
34	Conventional adenoma	14	+75%; ++25% (H score = 125)	+60%; ++30% (H score = 120)
35	Conventional adenoma	9	++3% (H score = 6)	+20% (H score = 20)
36	Oncocytic adenoma	21	+10%; ++5% (H score = 20)	+70%; ++20%; +++10% (H score = 140)
37	Oncocytic adenoma	18	++90%; +++10% (H score = 210)	+70% (H score = 70)
38	Conventional adenoma	16	No expression in adenoma cells (H score = 0)	+5% (H score = 5)
39	Conventional adenoma	19	+70%; ++20% (H score = 110)	+50% (H score = 50)
40	Oncocytic adenoma	11	+2% (H score = 2)	+50% (H score = 50)
41	Oncocytic adenoma	15	+40%; ++10% (H score = 60)	+5% (H score = 5)
42	Lipoadenoma	12	+50%; ++30% (H score = 110)	NA
43	Water-clear cell adenoma	19	+20%; ++70%; +++10% (H score = 190)	+70% (H score = 70)
44	Conventional adenoma	15	+70%; ++30% (H score = 130)	NA
45	Conventional adenoma	25	+20%; ++20% (H score = 60)	++50% (H score = 100)
46	Oncocytic adenoma	8	+20%; ++60%; +++10% (H score = 170)	++80% (H score = 160)
47	Conventional adenoma	33	+5%; ++3% (H score = 11)	NA
48	Conventional adenoma	37	++90%; +++10% (H score = 210)	++100% (H score = 200)
49	Conventional adenoma	18	+30%; ++40%; +++10% (H score = 140)	+70%; ++10% (H score = 90)
50	Conventional adenoma	20	+40%; ++60% (H score = 160)	++50% (H score = 100)
51	Conventional adenoma	35	+30%; ++60%; +++10% (H score = 180)	++80% (H score = 160)
52	Conventional adenoma	12	+50%; ++30%; +++10% (H score = 140)	++70% (H score = 140)
53	Conventional adenoma	15	+30%; ++20%; +++10% (H score = 100)	+50% (H score = 50)
54	Conventional adenoma	16	No expression in adenoma cells (H score = 0)	++20% (H score = 40)
55	Conventional adenoma	10	++20%; +++80% (H score = 280)	+30% (H score = 30)
56	Conventional adenoma	18	+5% (H score = 5)	+20% (H score = 20)

Note: Case-level data are shown for completeness and transparency. The water-clear cell adenoma (*n* = 3, Nos. 10, 21, 43) and lipoadenoma (*n* = 1, No. 42) subtype rows are purely descriptive; with such small subgroup sizes, these values do not support statistical comparison between subtypes and are summarized separately, with individual case data in Supplementary Table S1. H-score, histochemical score. Staining intensity: +, weak; ++, moderate; +++, strong. H-score categories: negative = 0; weak = 1–100; moderate = 101–200; strong = 201–300. Percentages indicate the approximate proportion of positive tumor cells in the evaluated compartment. The symbols record the raw staining intensity, whereas the H-score category is derived from the calculated intensity × percentage product.

## Data Availability

The data presented in this study are available from the corresponding author upon reasonable requests.

## Supplementary Materials

The following supporting information can be downloaded at: https://www.mdpi.com/article/10.3390/cancers18183026/s1. Table S1: Case-level integrin αvβ6 immunohistochemical data for the water-clear cell adenoma (*n*  =  3) and lipoadenoma (*n*  =  1) subgroups.

## References

[1] Silver Karcioglu A.L., Triponez F., Solórzano C.C., Iwata A.J., Abdelhamid Ahmed A.H., Almquist M., Angelos P., Benmiloud F., Berber E., Bergenfelz A. (2023). Emerging Imaging Technologies for Parathyroid Gland Identification and Vascular Assessment in Thyroid Surgery: A Review from the American Head and Neck Society Endocrine Surgery Section. JAMA Otolaryngol. Head. Neck Surg..

[2] Westerdahl J., Bergenfelz A. (2004). Sestamibi scan-directed parathyroid surgery: Potentially high failure rate without measurement of intraoperative parathyroid hormone. World J. Surg..

[3] Lalonde M.N., Correia R.D., Syktiotis G.P., Schaefer N., Matter M., Prior J.O. (2023). Parathyroid Imaging. Semin. Nucl. Med..

[4] Scerrino G., Paladino N.C., Graceffa G., Melfa G., Orlando G., Di Vuolo R., Lo Cicero C., Murabito A., Radellini S., Richiusa P. (2025). Primary Hyperparathyroidism: 18F-Fluorocholine PET/CT vs. 4D-CT for Parathyroid Identification: Toward a Comprehensive Diagnostic Framework—An Updated Review and Recommendations. J. Clin. Med..

[5] Gulati S., Chumber S., Puri G., Spalkit S., Damle N.A., Das C.J. (2023). Multi-modality parathyroid imaging: A shifting paradigm. World J. Radiol..

[6] Chakrabarty N., Mahajan A., Basu S., D’Cruz A.K. (2024). Imaging Recommendations for Diagnosis and Management of Primary Parathyroid Pathologies: A Comprehensive Review. Cancers.

[7] Treglia G., Trimboli P., Huellner M., Giovanella L. (2018). Imaging in primary hyperparathyroidism: Focus on the evidence-based diagnostic performance of different methods. Minerva Endocrinol..

[8] Morris M.A., Saboury B., Ahlman M., Malayeri A.A., Jones E.C., Chen C.C., Millo C. (2021). Parathyroid Imaging: Past, Present, and Future. Front. Endocrinol..

[9] Meecham A., Marshall J.F. (2020). The ITGB6 gene: Its role in experimental and clinical biology. Gene X.

[10] Breuss J.M., Gallo J., DeLisser H.M., Klimanskaya I.V., Folkesson H.G., Pittet J.F., Nishimura S.L., Aldape K., Landers D.V., Carpenter W. (1995). Expression of the beta 6 integrin subunit in development, neoplasia and tissue repair suggests a role in epithelial remodeling. J. Cell Sci..

[11] Niu J., Li Z. (2017). The roles of integrin αvβ6 in cancer. Cancer Lett..

[12] Steiger K., Quigley N.G., Groll T., Richter F., Zierke M.A., Beer A.J., Weichert W., Schwaiger M., Kossatz S., Notni J. (2021). There is a world beyond αvβ3-integrin: Multimeric ligands for imaging of the integrin subtypes αvβ6, αvβ8, αvβ3, and α5β1 by positron emission tomography. EJNMMI Res..

[13] Kuyumcu S., Denizmen D., Has-Simsek D., Poyanli A., Uzum A.K., Buyukkaya F., Isik E.G., Onder S., Aksakal N., Ozkan Z.G. (2024). (68)Ga-Trivehexin PET/CT: A promising novel tracer for primary hyperparathyroidism. Eur. J. Nucl. Med. Mol. Imaging.

[14] Güzel F., Kömek H., Alan N., Can C., Güven M., Akçay K., Güzel Y., İpek F.K., Kılıç R., Beydağı G. (2026). Beyond conventional maging: The diagnostic performance of [(68)Ga]Ga-Trivehexin PET/CT for detecting hyperfunctioning parathyroid tissue in primary hyperparathyroidism. Ann. Nucl. Med..

[15] Kimura R.H., Iagaru A., Guo H.H. (2023). Mini review of first-in-human integrin αvβ6 PET tracers. Front. Nucl. Med..

[16] Erickson L.A., Mete O., Juhlin C.C., Perren A., Gill A.J. (2022). Overview of the 2022 WHO Classification of Parathyroid Tumors. Endocr. Pathol..

[17] Badiu H.C. (2024). WHO Classification of Endocrine and Neuroendocrine Tumors. WHO Classification of Tumors.

[18] Tuffaha M., Hananeh W., Starke M. (2026). Immunohistochemical Evaluation of Integrin αvβ6 Expression in Gastric and Gastroesophageal Junction Adenocarcinoma as a Histopathology-Driven Biomarker for Integrin αvβ6-Targeted Radiotheranostics. Cancers.

[19] Dimitrakopoulou-Strauss A., Pan L., Sachpekidis C. (2023). Long axial field of view (LAFOV) PET-CT: Implementation in static and dynamic oncological studies. Eur. J. Nucl. Med. Mol. Imaging.

[20] Vogel W.V., Steemers-de Boer M.M., Jansen S.M., Rijkhorst E.J., de Wit-van der Veen B.J., van der Hiel B., Braat A. (2025). Clinical benefits of LAFOV PET/CT in growing demand for molecular imaging. Eur. J. Nucl. Med. Mol. Imaging.

[21] Alberts I., Sari H., Mingels C., Afshar-Oromieh A., Pyka T., Shi K., Rominger A. (2023). Long-axial field-of-view PET/CT: Perspectives and review of a revolutionary development in nuclear medicine based on clinical experience in over 7000 patients. Cancer Imaging.

[22] Kong S.H., Kim J.H., Kim S.W., Shin C.S. (2021). Radioactive Parathyroid Adenomas on Sestamibi Scans: Low Parathyroid Hormone Secretory Potential and Large Volume. Endocrinol. Metab..

[23] Wang Y., Liu Y., Li N., Xu K., Zhang W. (2023). Quantitative application of dual-phase 99mTc-sestamibi SPECT/CT imaging of parathyroid lesions: Identification of optimal timing in secondary hyperparathyroidism. EJNMMI Phys..

[24] Elgazzar A.H., Anim J.T., Dannoon S.F., Farghaly M.M. (2017). Ultrastructure of Hyperfunctioning Parathyroid Glands: Does it Explain Various Patterns of (99m)Tc-sestamibi Uptake. World J. Nucl. Med..

[25] Huellner M.W. (2026). [18F] Fluorocholine PET/MR Imaging. Parathyroid Imaging.

[26] Urso L., Napolitano R., Speltri G., Tuncel M., Badrane I., Uccelli L., Porto F., Martini P., Niorettini A., Cittanti C. (2025). (68)Ga-Trivehexin: Current Status of αvβ6-Integrin Imaging and Perspectives. Cancers.

[27] Tuffaha M., Hananeh W., Bangemann N., Tuffaha A., Starke M. (2026). Immunohistochemical Evaluation of Integrin β6 Expression in Triple-Negative Breast Cancer as a Predictive Biomarker for Therapeutic and Diagnostic Radionuclides. Biomolecules.

[28] Ganguly T., Bauer N., Davis R.A., Foster C.C., Harris R.E., Hausner S.H., Roncali E., Tang S.Y., Sutcliffe J.L. (2023). Preclinical Evaluation of (68)Ga- and (177)Lu-Labeled Integrin α(v)β(6)-Targeting Radiotheranostic Peptides. J. Nucl. Med..

[29] Movahedi M.M., Karimaghaei G.R., Noori A., Atefi M., Mahmoudi T., Gheisari F. (2022). Changes in Serum Alkaline Phosphatase, Calcium, and Parathyroid Hormone with Different Doses of Iodine Therapy. Galen. Med. J..

[30] Sathe S.V., Sparkman B., Bernard E., Smith E.R., He K., Chiu A., Brown T.C. (2025). Presentation and Surgical Outcomes of Primary Hyperparathyroidism after Radioactive Iodine Therapy. J. Surg. Res..

[31] Tuffaha M., Hananeh W., Shiban E., Starke M. (2026). Integrin αvβ6 Expression in the Human Pituitary Gland and Pituitary Neuroendocrine Tumors: Immunohistochemical Characterization with Potential Relevance to αvβ6 PET/CT Pituitary Uptake and Theranostic Implications. Biomolecules.

