# Supplementary material for: Integrin αvβ6 Expression in Parathyroid Adenomas and Adjacent Non-Neoplastic Parathyroid Tissue: Immunohistochemical Rationale for Integrin αvβ6-Targeted PET Imaging
Source: Cancers (Basel). 2026 Sep 17;18(18):3026. doi: 10.3390/cancers18183026 (PMC13604767; doi:10.3390/cancers18183026)
Supplement: Supplementary file 1 [file cancers-18-03026-s001.zip › cancers-4571914-supplementary.pdf]

Table S1. Case-level integrin αvβ6 immunohistochemical data for the water-clear cell adenoma (n = 3) and lipoadenoma (n = 1) subgroups.

No.	Parathyroid adenoma / subtype	Dimension (mm)	Integrin αvβ6 expression in parathyroid adenoma	Integrin αvβ6 expression in adjacent parathyroid tissue
10.	Water-clear cell adenoma	18	+10%; ++5%<br>(H score= 20)	++10%<br>(H score= 20)
21.	Water-clear cell adenoma	15	No expression in adenoma cells<br>(H score= 0)	+30%<br>(H score= 30)
43.	Water-clear cell adenoma	19	+20%; ++70%; +++10%<br>(H score= 190)	+70%<br>(H score= 70)
42.	Lipoadenoma	12	+50%; ++30%<br>(H score= 110)	NA
